# Ultra-Light Reduced Graphene Oxide Based Aerogel/Foam Absorber of Microwave Radiation

**DOI:** 10.3390/ma12020213

**Published:** 2019-01-10

**Authors:** Artyom Plyushch, Tianliang Zhai, Hesheng Xia, Chiara Santillo, Letizia Verdolotti, Marino Lavorgna, Polina Kuzhir

**Affiliations:** 1 Institute for Nuclear Problems of Belarusian State University, Bobruiskaya 11, 220030 Minsk, Belarus; artyom.plyushch@gmail.com; 2 Guizhou Building Material Quality Supervision Testing Center, Guiyang 550000, China; zhaitlwork@163.com; 3 State Key Laboratory of Polymer Materials and Engineering, Sichuan University, Chengdu 610065, China; xiahs@scu.edu.cn; 4 Institute of Polymer, Composites and Biomedical Materials, IPCB-CNR, Naples 80125, Italy; csantillo@unina.it (C.S.); letizia.verdolotti@cnr.it (L.V.); mlavorgn@unina.it (M.L.); 5 Radio Engineering Department, Tomsk State University, 36 Lenin Prospekt, 634050 Tomsk, Russian

**Keywords:** reduced graphene oxide, polyurethane foam, aerogel, microwave, absorption, electromagnetic interference shielding, compressive deformation

## Abstract

We present the polarization-dependent highly absorptive in Ka-band composition of conventional polyurethane foam filled with in situ synthesized aerogel coated by reduced graphene oxide (rGO). The rGO-based aerogel was in situ prepared into the open-cell polyurethane foam (PUF) skeleton through a bidirectional freeze-drying process. The aerogel is composed of the flat lamellas stacks, possessing the anisotropic structure and unique electromagnetic properties. Further improvement of the electromagnetic shielding ability was possible by the rGO coating introduction as a coupling layer between PUF and rGO-based aerogel. This enhances the overall conductivity of the resulting composites: 1.41 + 3.33*i* S/m vs. 0.9 + 2.45*i* S/m for PUF loaded with in situ synthesized aerogel without rGO coating.With this mechanically robust plane easy to process coating one could achieve −20 dB by power with the record light structure (0.0462 g/cm^2^). That could compete in view of the weight per cm^2^ even with graphene-based absorbers comprising either dielectric matching elements or back metal reflectors, or both.

## 1. Introduction

Carbon porous structures are known as effective ultra-light electromagnetic shields [1,2,3,4,5,6,7,8,9,10,11,12,13]. However, in most of the cases foams, aerogels, xerogels, and periodic architectures made of highly conductive glassy carbon skeleton (the conductivity is in the range from a few thousands to a few tens of thousands S/m) are highly reflective in microwave range. This is because all their characteristic geometrical parameters, such as cells and windows sizes, are much smaller than the microwave wavelength. Therefore, they could be presented schematically as a homogeneous conductive bulk structure which conductivity in non-resonant regime is roughly proportional to the “foam” density. It has been recently shown [13] that highly conductive skeleton is responsible for the sharp resonance absorption peaks at given frequencies (in most cases in THz frequency ranges), which position corresponds to the cell and/or window size of glassy carbon-based meshes.

When we are looking for highly absorptive structures in wide frequency range intuitively, it is clear that one needs to approach to as better as possible matching of shielding component with a free space. The best matching could be achieved via either so called Salisbury screen (the thickness of the absorptive dielectric should correspond to 1/4 of the electromagnetic wavelength) [14,15,16] or by cellular structure composed of not so highly conductive as glassy carbon skeleton in order to suppress reflection. In that sense, 3D printed components combining dielectric matrix and carbon containing conductive filament with conductivity at the level of a few tens of S/m look very attractive [17]. Although 3D printed layered structures are efficient (even if not perfect) absorbers of microwave radiation, they are relatively heavy in comparison with carbon porous monoliths [13] or graphene-based absorbers on the top of appropriate dielectric layer [18,19,20], as well as metamaterials like epsilon-near-zero substrate [21] or back reflector [22].

An interesting alternative to all the mentioned options could be provided by the structure combining the light (density 0.065 g/cm^3^) polymer foam with conductive ultra-light aerogel (density 0.0081 g/cm^3^), which original synthesis route has been recently proposed in our papers [23,24].

The present work will (i) study the electromagnetic properties of these polyurethane foams (PUF) and reduced graphene oxide (rGO) coated PUF loaded with in situ synthesized aerogel embedding rGO; and (ii) advertise them as one of the newly developed lightest absorbers of microwave radiation that demonstrates an outstanding mechanical robustness against compression deformations. The rGO-based aerogel was in situ prepared into the open cell PUF skeleton through a bidirectional freeze-drying process [25]. The obtained aerogel consisted of the flat lamellas stacks, which possessed the anisotropic structure and unique electromagnetic properties. In order to further improve the electromagnetic properties, the rGO coating was used as coupling layer between PUF and rGO-based aerogel to enhance the overall conductivity of the resulting composites. Chitosan (CS) was chosen to prepare rGO-based aerogel because it can stabilize the GO in suspensions thus allowing the formation of reliable aerogels [26] and because the -NH_2_ groups on the CS macromolecular chains can promote the thermal reduction of GO [27].

Being broadband absorber in Ka-band (26–37 GHz) investigated ultra-light structures could be utilized for many practical applications, solving a number of hot problems such as electromagnetic security in space, widespread absorbers in secure electronic schemes suppressing re-scattered and crosstalk signals, anti-scanning/cloaking coatings and microwave sensors to detect and decode low intensity microwave signals.

## 2. Sample Preparation

### 2.1. Materials

Flake graphite (~75μm) was purchased from Qingdao Tianhe Graphite Co. Ltd. (Qingdao, China). Potassium permanganate (KMnO_4_) were purchased from Chengdu Kelong Chemical Reagent Company (Chengdu, China). Hydrochloric acid (HCl) and concentrated sulfuric acid (H_2_SO_4_), which were all analytical-grade, were purchased from Sichuan Xilong Chemical Co. Ltd. (Chengdu, China). Medium molecular weight chitosan (448877 Aldrich with a deacetylation degree greater than 75–85%), ascorbic acid, acetic acid (ACS reagent, ≥99.7%) and silver paste were purchased from Sigma-Aldrich (Milan, Italy).

Polyurethane foams were prepared by using Methylene diphenyl diisocyanate (MDI), SpecflexNE 134 (isocyanate index 0.8) and synthetic polyol, SpecflexNF 660 (hydroxyl value: 65.3–75) kindly provided by Dow chemicals, Italy, and Bio-based polyol, FF1BiosucciniumTM, (hydroxyl value equal to 61.5 mgKOH/g) kindly provided by Reverdia, Netherlands. CH_3_COOK, L6164, PM40 (kindly provided by Momentive, Italy) and distilled water were used as catalyst, surfactant, and blowing agent, respectively.

### 2.2. Preparation of GO and GO/CS Solution

The preparation of graphene oxide was described in our previous work [23]. The GO/CS solution (5/5 mg/mL) was prepared by mixing CS solution (10 mg/mL, 40 mL) and GO solution (10 mg/mL, 40 mL) in a 100 mL centrifuge tube under 30 min probe-sonication treatment. The CS solution (10 mg/mL) was prepared by dissolving CS powder (10 g) into 1000 mL of 1 v/v% acetic acid solution and stirred for 12 h at room temperature. A uniform GO suspension (10 mg/mL) was obtained by dispersing GO powder (0.4 g) into deionized water (40 mL) and sonicated in a bath-sonicator for 2 h.

### 2.3. Preparation of PUFs and rGO-Coated PUFs

Open-cell polyurethane foams were prepared by adding MDI (38.6 g) to a mixture of polyols (i.e., bio-based polyol/synthetic polyol, 10 g/40 g), CH_3_COOK (0.1 g), PM40 (0.1 g) and L6164 (0.1 g) and H_2_O. After stirring for about 20 s, the mixture was quickly poured into a mold. The resulting polyurethane foams were post-cured in oven at 75 °C for 1 h, and 120 °C for further curing for 3 h, respectively.

The reduced graphene oxide-coated PUF foams (rGO-PUF) were prepared by deposition of in situ thermal reduced graphene oxide nanoplatelets onto the surfaces of polyurethane foams. First, the GO/ascorbic acid premixed solution (50 mL) was filled into a centrifuge tube (50 mL) containing PUF samples (15 mm × 15 mm × 15 mm) under vacuum. The GO/ascorbic acid premixed solution was prepared by mixing GO (100 mg) and ascorbic acid (4000 mg) into 100 mL DI water. Second, the centrifuge tube containing PUF and GO/ascorbic acid solution was transferred into an oven at 90 °C for 2 h to in-situ reduce the GO and let the rGO deposited on the PUF surfaces. Finally, the resulting rGO-PUF samples were washed with ethanol and deionized water, and dried in an oven at 90 °C for an additional 3 h.

### 2.4. Preparation of Graphene-Based Chitosan Aerogel/PUF Composites by Bidirectional Freeze-Drying

First, the GO/CS solution (5/5 mg/mL, 50 mL) was filled into a centrifuge tube (50 mL) containing three pieces of PUF cubes (15 mm × 15 mm × 15 mm) under the action of vacuum. The centrifuge tube with three pieces of PUF cubes was placed in a vacuum desiccator with an 80 kPa below atmospheric pressure in advance. Then, we kept the vacuum constant for about 30 min for degassing. Second, the PUF cubes filled with GO/CS solution were carefully transferred into a silicone rubber mold placed on the top of a steel plate for bidirectional freezing. One end of the steel plate was immersed in liquid nitrogen to induce a temperature gradient on the plate surface. When the samples were completely frozen, they were removed from the mold and lyophilized to remove the ice phase. Finally, the obtained aerogel/PUF composites were thermally dried for removing the residual acetic acid and then thermally treated at 200 °C for 12 min to promote the thermal reduction of the GO/CS aerogel. Graphene-based aerogel/rGO-PUF samples were produced with a similar procedure by starting from polyurethane foams coated with reduced graphene oxide (rGO-PUF). More details about the preparation of all samples are provided in publications by our research team [24].

## 3. Experimental

The microwave measurements (26–37 GHz frequency range, Ka-band) were carried out using scalar network analyzer R2-408R, (ELMIKA, Vilnius, Lithuania) by means of the waveguide method. Samples were accurately cut in parallelepiped shape in order to fit the waveguide cross section of 7.2 × 3.4 mm. Samples thickness was chosen as 2.3 mm in order to comprise at least five pores (typical pore size was estimated as approx. 500 microns). The electromagnetic (EM) responses were measured as ratios of transmitted/input (S_21_) and reflected/input (S_11_) signals. Absorption was re-calculated as A = 1 − R − T = 1 − (S_21_)^2^ − (S_11_)^2^.

The compressive properties of the materials were tested by using an Instron (5564) universal testing instrument. The specimens (40 mm × 40 mm × 10 mm) were compressed with a 3 mm/min strain rate along the shortest direction.

The results of Thermogravimetric analysis (TGA) can be found in Supplementary materials.

## 4. Results and Discussion

### 4.1. Structural Characterization

**Figure 1:**
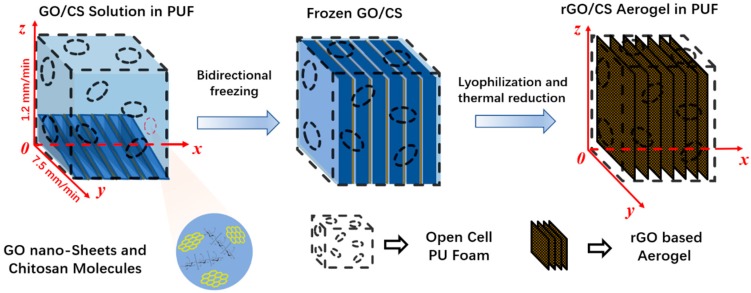
Schematic illustrations of the fabrication of rGO/CS aerogel/polyurethane foam (PUF) composite.

The aerogel/foam composites were prepared by the in situ growth of rGO/CS aerogel inside the PUF through bidirectional freeze-drying method, which is schematically illustrated in Figure 1. 3D coordinates were established according to the ice growth direction. The freezing starting line is determined as the *x*-axis. The vertical ice growth direction with the growth rate of 1.2 mm/min is defined as the *z*-axis, and the horizontal ice growth direction with the growth rate of 7.5 mm/min is defined as the *y*-axis.

**Figure 2:**
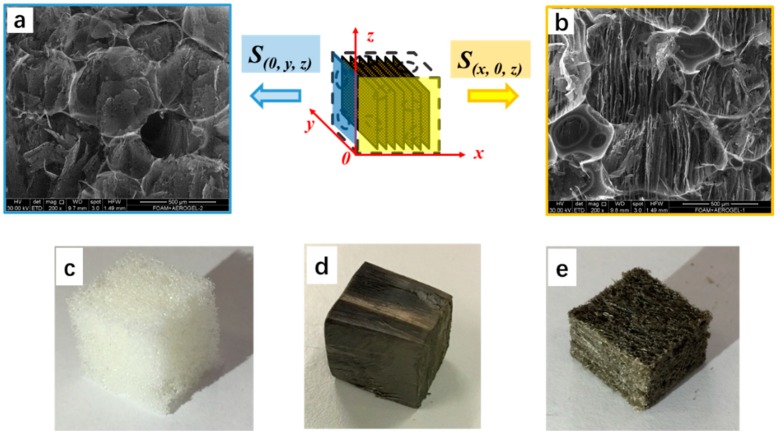
Scanning electron microscope (SEM) images to show (**a**) the flat wall structure of the aerogel/PUF composites observed in the surface of S (0, *y*, *z*) and (**b**) the aligned lamellas structure observed in S (*x*, 0, *z*). Optical photos of (**c**) the pristine PUF, (**d**) the outside surface and (**e**) the cross section of the aerogel/foam composite.

Figure 2 shows schematic illustration of the resulting aerogel/PUF composite with 3D coordinates. Comparing the coordinates established in Figure 1, the coordinates were rotated to show the S (*x*, 0, *z*) and S (0, *y*, *z*) surfaces. The parallel lamellas structure of the aerogel/PUF composite can be clearly seen when the composite was observed from S (*x*, 0, *z*) surface, Figure 2b; while, the flat wall structure was obtained when the S (0, *y*, *z*) surface was observed, Figure 2a. The photographs of pristine PUF, the outside surface and the cross section of the aerogel/foam composite are presented in Figure 2c–e, correspondently.

**Figure 3:**
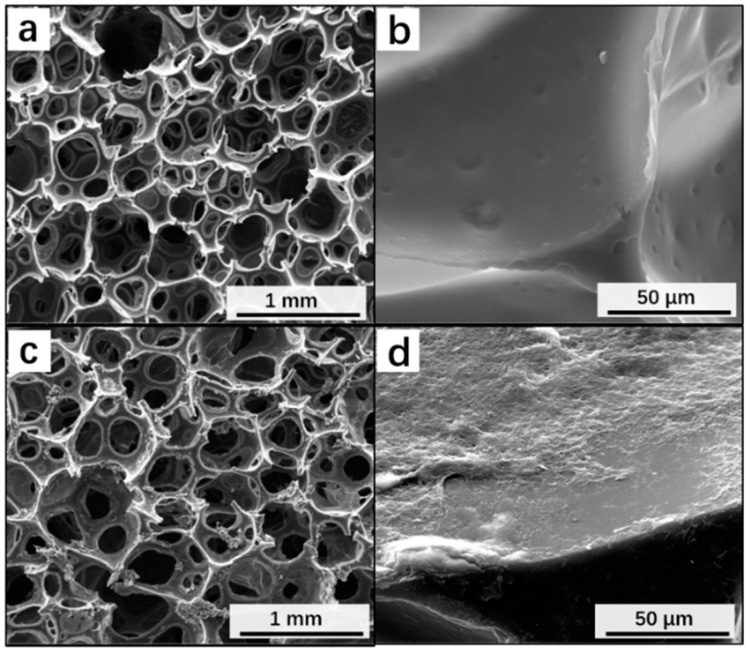
SEM images to show the porous structure of the (**a**) pristine PUF and (**c**) rGO coated PUF. High magnification SEM images to show the (**b**) smooth surface of the pristine PUF and (**d**) rough rGO coating layer on the surface of rGO coated PUF.

According to Figure 3 pore size of the PUF was estimated as 503 ± 76 μm.

**Figure 4:**
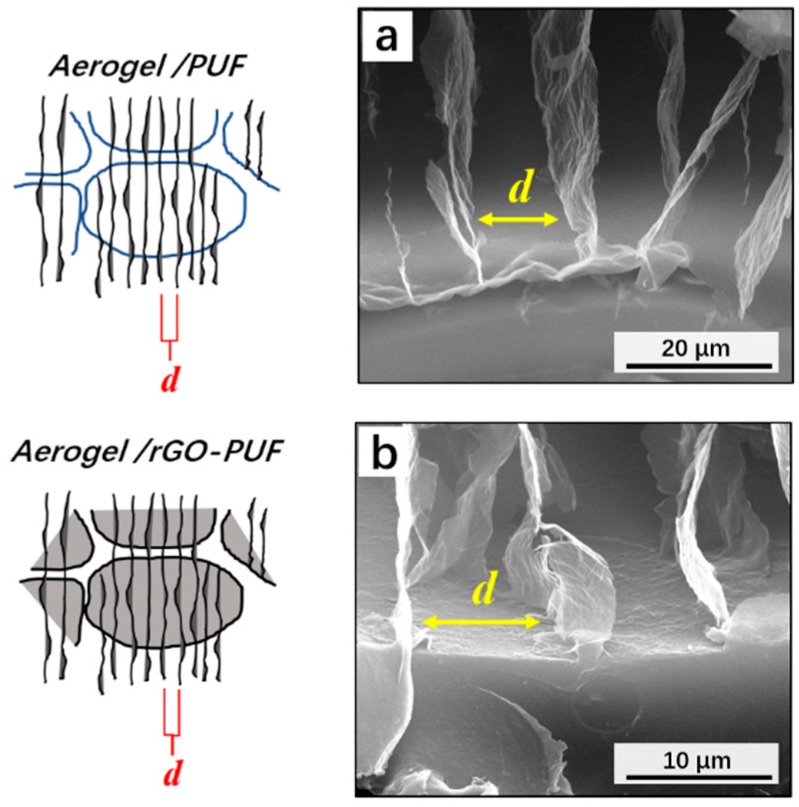
SEM images to show the aligned lamellas structure of the aerogel and the bonding interface of the (**a**) aerogel/PUF composite and (**b**) aerogel/rGO-PUF composite.

Figure 4 presents the distance between two aerogel’s walls as 12.4 ± 2.8 μm.

### 4.2. Microwave Probing

Taking into account the fact that the wavelengths range corresponding to the studied microwave frequencies 26–37 GHz are 8–11 mm, obviously the characteristic distances for microwaves at least in (0, *y*, *z*) plane are much higher than the sample morphologic elements (both pores and lamellas). Microwave frequencies the interaction of the PUF, as well as aerogel/PUF and aerogel/rGO-PUF composites could be described by macroscopic parameters like conductivity or complex dielectric permittivity.

In fact, the foam-like porous structure can be roughly considered as the composite of air and the skeleton as the second phase inclusion, percolated in all three directions. Since the material is considered as homogeneous composite, the Fresnel formula may be applied for the calculation of S-parameters:

(1) $$S_{11}=\frac{-2sin\left(\gamma \tau \right)\left(\gamma_{0}^{2}-\gamma^{2}\right)}{sin\left(\gamma \tau \right)\left(\gamma_{0}^{2}-\gamma^{2}\right)+2i\gamma \gamma_{0}cos\left(\gamma \tau \right)},$$

(2) $$S_{21}=\frac{2\gamma /\gamma_{0}}{\frac{-2\gamma }{\gamma_{0}}cos\left(\gamma \tau \right)+i\left({\left(\frac{\gamma }{\gamma_{0}}\right)}^{2}+1\right)sin\left(\gamma \tau \right)},$$

where $\gamma =\sqrt{{\left(\frac{2\pi }{\lambda }\right)}^{2}\varepsilon -{\left(\frac{\pi }{a}\right)}^{2}}$,$\gamma_{0}=\sqrt{{\left(\frac{2\pi }{\lambda }\right)}^{2}-{\left(\frac{\pi }{a}\right)}^{2}}$, τ is the sample’s thickness, a = 7.2 mm is the width of waveguide, and ε is the complex dielectric permittivity of the investigated sample.

**Figure 5:**
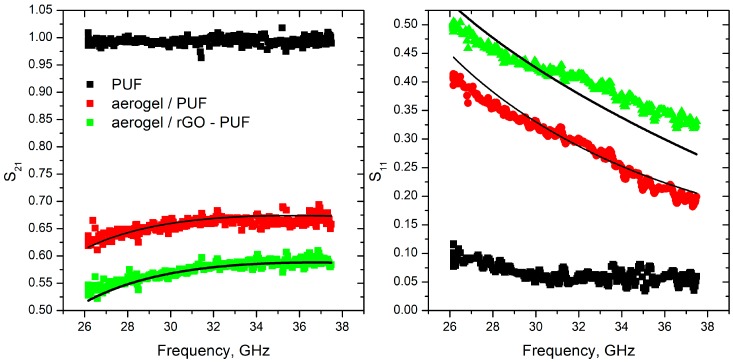
S-parameters of studied foams in microwave range. Symbols correspond to the measured data, lines are modeling results.

Measured and modeled S-parameters of studied samples are presented in Figure 5.

As one can see, the pure polyurethane foam is fully transparent for the microwave radiation and does not provides any impact on the transmitted (S_21_) signal, while modified foams transmit only 25–40% of the initial radiation. In contrast, the conductivity of tannin and polyurethane template-based carbon foams [5,10,11,13] could be described as the conductivity of metal-type material by a simple formula:

(3) $$\varepsilon \left(\omega \right)=1+\frac{i\sigma }{\omega \varepsilon_{0}},$$

the conductivities of modified PUF, both aerogel/PUF and aerogel/rGO-PUF, are complex values *σ*(*ω*) = *σ*′(*ω*) + *iσ*″ (*ω*), and thegeneral formula $\varepsilon \left(\omega \right)=\frac{\sigma }{i\omega \varepsilon_{0}}$ should be used.

Comparing the best fit (lines in Figure 5) calculated using Equations (1) and (2) and measured data (symbols in Figure 5), the conductivity of the modified foams has been reconstructed as σ = 1.41 + 3.33*i* S/m and 0.9 + 2.45*i* S/m for aerogel/rGO-PUF and aerogel/PUF respectively.

**Figure 6:**
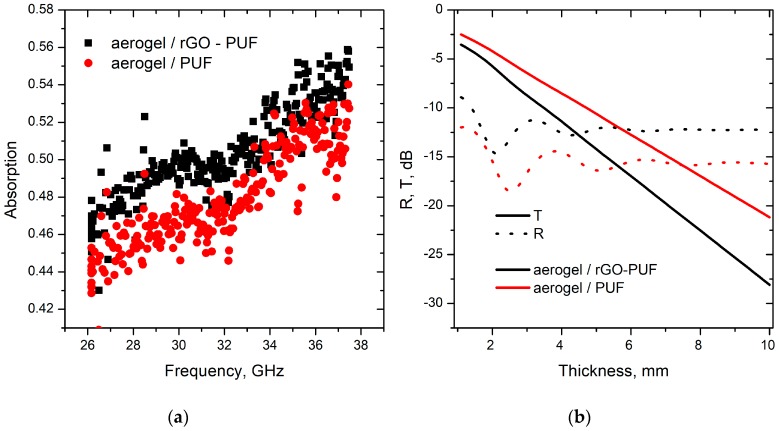
(**a**) Measured absorption spectra of loaded foams in microwave range for 2.3 mm thick samples. (**b**) Calculated transmission (T) and reflection (R) of the studied samples as a function of thicknesses.

The absorption spectra of loaded foams are demonstrated in Figure 6a for 2.3 mm thick layer of aerogel/PUF samples. Both aerogel/PUF and aerogel/rGO-PUF show high absorption ability (up to 56% of initial radiation), having extremely low density (0.065 g/cm^3^ and 0.066 g/cm^3^ for Aerogel/PUF and Aerogel/rGO-PU correspondently). This means that both samples are really perspective candidates for producing lightweight electromagnetic absorbers for microwaves, in favor of aerogel/rGO-PUF.

The calculated electromagnetic interference shielding efficiency (T = (S_21_)^2^, R = (S_11_)^2^) in dB is presented in Figure 6b vs samples thickness. One arrives to the state-of-the-art figures of merits for Ka-band, i.e., −20 dB, for 7 mm thick aerogel/foam, being 0.00462 g/cm^2^. That is the given porous structures could compete even with a graphene based absorber, as those surface densities should include also the density of matching conventional dielectrics, polymer spacers, and/or back reflectors.

### 4.3. Anisotropy Study

**Figure 7:**
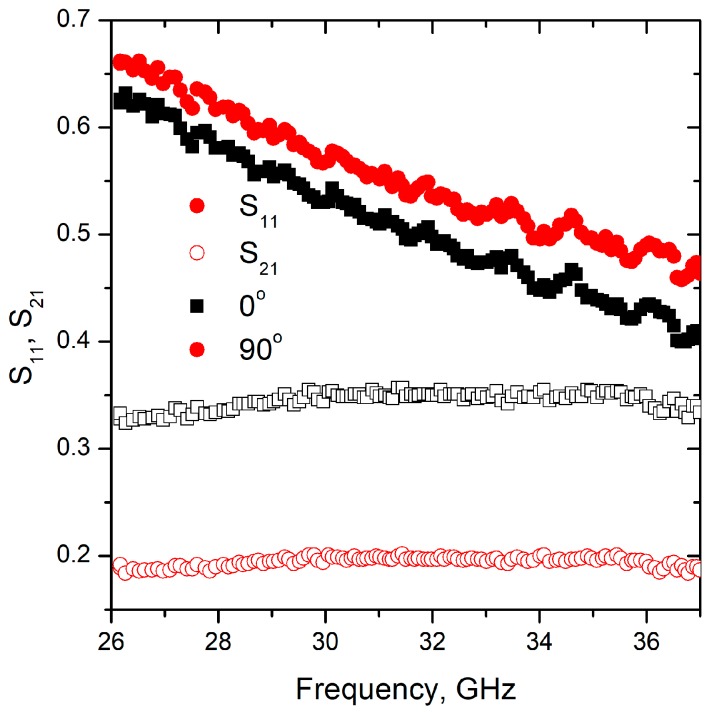
S-parameters of the aerogel/rGO-PUF with different orientation of the structure elements to incident irradiation polarization.

For the anisotropic properties measurements two different types of samples were studied, one was oriented with lamellas in parallel to the electric field (0, *y*, *z*) plane, and another one had aperpendicular orientation (*x*, 0, *z*) plane. The results for aerogel/rGO-PUF are presented in Figure 7.

In contract to previously collected data for other types of isotropic rGO based aerogels (see [28,29]), significant difference was observed in electromagnetic response of aerogel/PUF of two different orientations; i.e., S_21_ was close to 0.2 vs. 0.33 for electric field parallel to the *z* and *x* direction, respectively. Moreover, there was no substantial difference in the reflection ability of the collected samples (S_11_ = 0.55 vs. 0.58 at 32 GHz), which corresponded to 30% and 33% of reflection. Thereby, namely absorption of aerogel comprising foams is sensitive to the polarization of electromagnetic field.

### 4.4. Mechanical Properties

**Figure 8:**
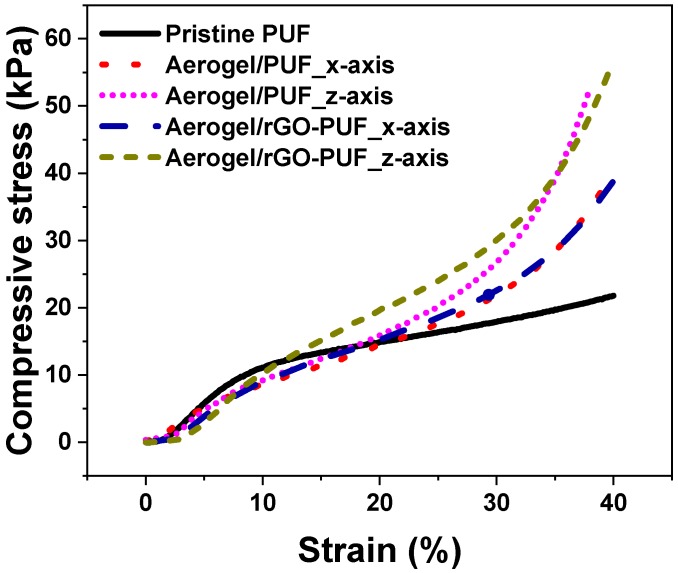
Compressive properties under 40% compressive strain.

The mechanical strength of rGO/CS aerogels prepared by freeze-drying methods is generally poor. The PUF plays the role of elastic scaffold to enhance the strength of the aerogel/PUF composites. Figure 8 shows that mechanical strength of both aerogel/PUF and aerogel/rGO-PUF composites is higher than that of pristine PUF. For instance, the compressive strength of the pristine PUF at 40% strain is 21.8 kPa while the strengths of the aerogel/PUF and aerogel/rGO-PUF composites compressive in *x*-axis at same strain are 40.3 and 38.9 kPa, respectively. As anisotropic materials, the composites possessed higher strength in the *z*-axis, which are 52.8 and 56.6 kPa for aerogel/PUF and aerogel/rGO-PUF composites at 40% strain, respectively. The aligned lamellas structure in the *z*-axis, Figure 2b, provide higher compression resistance than the parallel lamellas in the *x*-axis. Compared to the aerogel/PUF composites, the rGO coating layer did not show obvious influence on the mechanical properties of the aerogel/rGO-PUF composites.

## 5. Conclusions

One of the lightest materials—polyurethane foam loaded with in situ synthesized aerogel in some cases coated with reduced graphene oxide—demonstrates an appropriate level of conductivity (*σ* = 1.41 + 3.33*i* S/m and 0.9 + 2.45*i* S/m) to provide a high level of electromagnetic interference shielding in Ka-band being only a few mm thick due to mostly absorption ability. According to TGA (see Supplementary materials), 12 min was chosen as the treatment time for thermal GO reduction, because satisfactory conductivity has been obtained at this treatment time (not too large to be fully reflective like glassy carbon made porous structures [13] or not too small like neat resin [11] to be fully transparent for microwave radiation). The prominent anisotropy of the electromagnetic response was observed: it is coursed by the polarization affected absorption of the studied shielding layers. With this record 0.00462 g/cm^2^ graphene oxide based coating one may achieve more than −20 dB of EMI SE for the simple plane of a mechanically robust structure for absorptive or anti-reflection microwave components applications.

## Supplementary Materials

The following are available online at http://www.mdpi.com/1996-1944/12/2/213/s1, Figure S1: Thermogravimetric curve of the GO/CS aerogel/PUF composite thermal treated under 200 °C for 2 h.
