# Supplementary material for: Ultra-Light Reduced Graphene Oxide Based Aerogel/Foam Absorber of Microwave Radiation
Source: Materials (Basel). 2019 Jan 10;12(2):213. doi: 10.3390/ma12020213 (PMC6356501; doi:10.3390/ma12020213)
Supplement: Supplementary file 1 [file materials-12-00213-s001.pdf]

materials

Article

Ultra-Light Reduced Graphene Oxide Based Aerogel/Foam Absorber of Microwave Radiation

Artyom Plyushch [1,†], Tianliang Zhai [2,†], Hesheng Xia [3], Chiara Santillo [4], Letizia Verdolotti [4], Marino Lavorgna [4] and Polina Kuzhir [1,5,*]

1 Institute for Nuclear Problems of Belarusian State University, Bobruiskaya 11, 220030 Minsk, Belarus; artyom.plyushch@gmail.com
2 Guizhou Building Material Quality Supervision Testing Center, Guiyang 550000, China; zhaitlwork@163.com
3 State Key Laboratory of Polymer Materials and Engineering, Sichuan University, Chengdu 610065, China; xiahs@scu.edu.cn
4 Institute of Polymer, Composites and Biomedical Materials, IPCB-CNR, Naples 80125, Italy; csantillo@unina.it (C.S.); letizia.verdolotti@cnr.it (L.V.); mlavorgn@unina.it (M.L.)
5 Tomsk State University, 36 Lenin Prospekt, 634050 Tomsk, Russian
* Correspondence: polina.kuzhir@gmail.com; Tel.: +375-29-605-1835
† These authors contributed equally to the paper.

Received: 29 November 2018; Accepted: 7 January 2019; Published: 10 January 2019

Supplementary materials

Thermal properties.

The thermogravimetric curve of the aerogel/PUF composite which was thermally treated at 200 °C for 2 h showing the weight losses of the composite during the thermal reduction process is presented in Figure 9. The weight loss is about 14.7% after 2 h at 200° C. The weight loss was mainly caused by the decomposition of the hydroxyl group on the GO nanosheet indicating that the effective thermal reduction of GO can be achieved. However, long treatment time could bring a lot of structural defects, which is harmful to the durability of the final composites. Hence, the 12 min was chosen as the treatment time for thermal reduction, because satisfactory conductivity has been obtained at this treatment time.

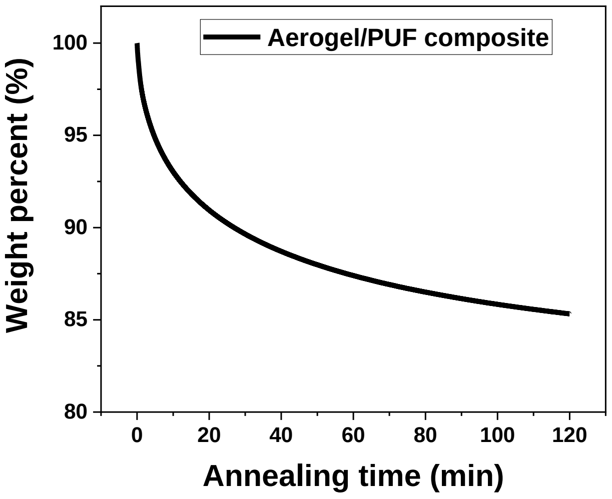

Figure S1. Thermogravimetric curve of the GO/CS aerogel/PUF composite thermal treated under 200 °C for 2 h.
